# Assessing Patient Satisfaction With the Discharge Process Through a Patient-Centered Lens

**DOI:** 10.7759/cureus.87923

**Published:** 2025-07-14

**Authors:** Angela T Nguyen, Janetta L Lam, Zachary M Schwartz, Abraham A Mascio, Emily Coughlin, Shanu Gupta

**Affiliations:** 1 Internal Medicine, University of South Florida Morsani College of Medicine, Tampa, USA; 2 Biostatistics, University of South Florida Morsani College of Medicine, Tampa, USA

**Keywords:** care team, hospital discharge, patient-centered care, patient satisfaction, provider communication

## Abstract

Introduction

The discharge process remains vital to ensuring patient satisfaction and understanding. Patient satisfaction is an important metric in determining hospital quality and has a correlation with better outcomes. Understanding discharge instructions and patient-reported readiness can also help reduce readmission rates. This study assessed the quality of the discharge process at a tertiary health care institute by measuring participant satisfaction using a locally developed quantitative Likert scale survey and a qualitative survey regarding discharge ease and care team communication.

Methods

A survey questionnaire was designed independently to address provider communication and patients’ understanding of discharge recommendations via a Likert scale. Both qualitative and quantitative questions were designed with guidance from previous studies in order to include components of successful discharge, including understanding of hospitalization reasons, follow-up appointments, medications, and overall discharge preparedness. Patients were recruited in a tertiary health care institute discharge lounge from the period of June 2023 to July 2023. The patient sample included adult patients who were waiting to be discharged in the lounge, excluding elective surgery admits or emergency room discharges. Data collection was performed by the authors of this study. Patient responses were analyzed for recurring themes regarding the stay and hospital discharge process, and statistical analysis was performed on quantitative data.

Results

From June 2023 to July 2023, 78 patients were interviewed using both the quantitative and qualitative surveys. Overall, participant-reported satisfaction with hospital stay ranged from five to 10 out of 10, with 49 (62.8%) participants rating the quality of care of their visit a 10/10. Mean satisfaction overall was 9.44 ± 0.996. Two questions exhibited a significant association with the participants’ overall quality rating of their stay: “I had adequate time talking to my physician” (p=0.031) and “I would return to this hospital for future healthcare needs” (p=0.01). No other questions were significantly associated with overall satisfaction. 55 (70.5%) participants reported having a primary care physician in the area, though this was not significantly associated with the level of agreement for any question. Qualitatively, some positive themes that emerged were the attentiveness of the care team and the efficiency of the discharge process. Some areas of improvement included logistical challenges in scheduling and ensuring bed availability, discharge procedures, and medication management, along with addressing issues related to inconsistent communication within the care team.

Conclusions

Overall, participant satisfaction with the discharge process was high, with decreased satisfaction for those who felt they did not have enough time with the physician. This idea is a clear starting point for producing a recommendation list, including items such as reducing wait times, enhancing patient-physician interaction, and standardizing institutional processes to boost patient satisfaction and post-discharge care quality. This recommendation list will encompass both quantitative and qualitative aspects, addressing various communication and logistical challenges to provide a holistic approach to improving the discharge process.

## Introduction

The discharge process from the hospital is the last step in a patient’s stay, and it provides the final point of interaction, helping patients transition back home and reducing the risk of readmission. Readmission within 30 days is related to poor clinical outcomes, with the Centers for Medicare & Medicaid Services reporting an overall readmission rate percentage of 14.7% among all Medicare beneficiaries in 2020 [1]. Indicative of care quality, readmission also results in higher spending by Medicare, costing billions of dollars yearly [1]. Although disparities exist between demographic, clinical, and geographic factors, interventions such as discharge planning and hospital quality improvement may improve readmission rates, not just among vulnerable populations [1]. Single interventions seem to be ineffective in preventing readmission [2]. A multifactorial approach addressing predictors of readmission, such as patient lack of understanding and preparedness, may mitigate readmission strain [3,4]. 

Past studies have demonstrated that the inability to list diagnoses and medications and limited medical knowledge overall contribute to difficulty with discharge plan adherence and worse outcomes [5, 6]. Physician communication plays a big role in understanding discharge instructions, and there is an overestimation of physicians' belief in understanding compared to patients [7]. Greater efforts on effective communication towards patient understanding of post-discharge care can contribute to a stronger perceived readiness for discharge, which correlates to greater satisfaction. Although there is mixed, weaker evidence on patient readiness and readmission, patient satisfaction could be correlated with lower readmission rates [8]. Attributes of readiness for hospital discharge have been identified as including elements of knowledge, communication, participation, and collaboration between the patient and the healthcare team [9, 10]. The goal of this study was to evaluate elements of the discharge process, specifically, patient understanding and satisfaction. Quality of discharge was assessed, focusing on understanding of follow-up and care, medications, and diagnoses as measures of effective care team communication and likelihood of future adherence. Patient satisfaction based on factors of diagnosis understanding and healthcare team involvement was also assessed to examine communication and perceived discharge readiness in the effectiveness of the discharge process. 

## Materials and methods

Convenience sampling was utilized to select participants. The study was performed at a single institution with over 1,000 inpatient beds. Patients utilizing the hospital departure lounge between June and July 2023 were invited to participate in the survey. The lounge sees about 100-115 patients daily. In total, 119 patients were interviewed for this study, 78 (65%) of whom were included in our analysis. Patients were excluded if admitted for elective surgery or discharged directly from the emergency room to capture an audience that had an unanticipated or longer stay, respectively, or were under 18. 

Informed consent was secured prior to questioning. Participants were asked a series of quantitative and qualitative questions regarding their hospital stay, discharge process, and other general medical information. These questions were developed and selected after an extensive literature review regarding other institutions’ discharge processes was examined [1, 2, 4, 5, 7, 10, 11]. Interviews were conducted orally and recorded using the Otter.ai app (Otter.ai, Inc., Los Altos, CA, USA). They were later transcribed for accuracy and analysis. The survey questions are provided in Appendix A. Quantitative survey answers were analyzed for the average mean rating from 0 to 10 on agreement with the statement. The independent-samples Kruskal-Wallis test was also used to analyze statement significance. Qualitative answers were examined for repeating themes and narratives surrounding the discharge process. 

Data collected from participants who chose to withdraw were included in our analysis. No additional data was collected after participant withdrawal. University of South Florida's Institutional Review Board (IRB) granted an exemption for this study.

## Results

Hospitalization reasons were collected and grouped into general categories, shown in Table 1. Reasons were categorized into general system complaints, providing an overview of discharge lounge patients. 

**Table 1:** Patient-reported reasons for hospitalization

Hospitalization reason	Frequency n(%)
Cardiopulmonary	18 (23.1%)
Trauma/musculoskeletal	12 (15.4%)
Infection/sickness	8 (10.3%)
Neurological	6 (7.7%)
Gastroenterology	16 (20.5%)
Other	18 (23.1%)

The quantitative survey data encompassed nine statements evaluating the quality of care received by patients during their hospital visit, including aspects such as understanding their diagnosis and rating the quality of care. Most patients expressed agreement or strong agreement with these statements. Notably, the highest mean rating, using a scale from 0 to 10 representing patients’ perceived strength of agreement, of 9.65 was observed for patients feeling prepared to leave the hospital. However, the statement assessing the adequacy of time spent talking to their physician had a lower mean rating of 8.25. 

Table 2 shows our survey respondents’ answers, with the significance of each statement relative to overall patient satisfaction with the discharge process displayed in the right-hand column. The significance of these statements was determined based on the independent-samples Kruskal-Wallis test. 

**Table 2:** Patients' perspectives on the discharge process and their strength of agreement with overall satisfaction

Statement	Did not answer n(%)	Strongly agree n(%)	Agree n(%)	Neutral n(%)	Disagree n(%)	Strongly sisagree n(%)	P-value
My questions were well answered prior to my discharge.	4 (5.1%)	31 (39.7%)	41 (52.6%)	1 (1.3%)	1 (1.3%)	0	0.075
I understand what appointment/follow-ups I need to go to next.	5 (6.4%)	27 (34.6%)	45 (57.7%)	1 (1.3%)	0	0	0.104
I understand how to take my medications once I leave the hospital.	7 (9%)	32 (41%)	39 (50%)	0	0	0	0.076
I am confident that I’ll be able to follow my discharge instructions at home.	7 (9%)	28 (35.7%)	43 (55.1%)	0	0	0	0.161
I understand my diagnosis/problem, I stayed in the hospital for.	7 (9%)	27 (34.6%)	42 (53.8%)	1 (1.3%)	1 (1.3%)	0	0.532
I feel prepared to leave the hospital.	5 (6.4%)	32 (41%)	38 (48.7%)	2 (2.6%)	0	1 (1.3%)	0.251
I had adequate time talking to my physician during my hospital stay.	5 (6.4%)	26 (33.3%)	39 (50%)	1 (1.3%)	6 (7.7%)	1 (1.3%)	0.031
I would return to this hospital for future healthcare needs.	6 (7.7%)	39 (50%)	29 (37.2%)	2 (2.6%)	2 (2.6%)	0	0.010

The statistical significance threshold for the hypothesis tests was set at 0.05, with asymptotic significance displayed throughout the analysis. In general, patients responded with general agreement in the three main categories surrounding our quantitative questions: understanding, confidence, and satisfaction. 

In assessing overall patient-reported satisfaction with their hospital stay, responses spanned a range of five to 10, with 62.8% of patients rating the quality of care at the highest level, 10/10. The mean overall satisfaction score was 9.44 ± 0.996. Two survey questions demonstrated a significant association with the participants' overall rating of their stay: "I had adequate time talking to my physician" (p=0.031) and "I would return to this hospital for future healthcare needs" (p=0.01). Conversely, no other survey questions exhibited significant associations with the overall satisfaction rating. Although 70.5% (n=55) of participants reported having a primary care physician (PCP) locally, this variable did not demonstrate a significant relationship with agreement levels for any of the survey questions. 

Further analysis utilizing independent-samples Kruskal-Wallis tests did not reveal significant differences in the distribution of ratings for the statement concerning adequate physician interaction across categories of feeling prepared to leave the hospital or feeling involved in their discharge process (p>0.05). 

In qualitative feedback, patients praised the attentiveness and positive attitudes of their care team, contributing positively to their hospital experiences, with one patient saying, “They try to stay on top of everything. You know when you have a nurse that comes in and checks even when she's not supposed to check? Yeah, that speaks volumes. Even if it's two seconds of their day to come in and they know you by name and say, Hey, [patient name], how are you this morning? It was a lot.” Additionally, many patients praised the “smoothness” of the discharge process, with one patient citing how the hospital has improved their discharge process already compared to his prior admission. 

Other patients, however, described frustration with logistical matters throughout their discharge process, with the most prominent concern being long delays with their discharge medications. One patient described how their daughter had been waiting for an extended period outside, while another said, “The first thing is that when they hand you your discharge paperwork, your medicine should already be processed, because I'm sitting here now… sitting waiting on my meds, you know.” Others expressed their frustration with doctors’ and the rest of the care team’s communication with one another, with one patient mentioning that their “timelines were kind of confusing.” The timelines for when things were going to happen were not very consistent with what was communicated.” 

## Discussion

The survey results reflect generally positive patient experiences and a sense of readiness for continued care while highlighting opportunities for enhancing physician-patient interaction to further improve overall care quality. This study uses a patient-centered lens to assess the discharge process, which provides a more comprehensive view of patient satisfaction. The use of both quantitative and qualitative measures can help bring a deeper understanding to the patient experience. A variety of hospitalization reasons were reported, which helps capture a more representative population. Patients reported a strong sense of preparedness leaving the hospital, which suggests positive outcomes regarding plan adherence. A lower rating was seen for the statement regarding adequate time talking to physicians, suggesting that the amount of time dedicated to patient-physician interaction may warrant improvement to enhance the overall quality of care provided to patients. However, “adequate” is poorly defined and may differ among both individual patients and physicians. For one university hospital study performed in Germany, physicians communicated for an average time of four minutes and 17 seconds per patient, out of an average 10-hour and 58-minute workday. Physicians in the hospital hold many responsibilities, and discussions with colleagues and documentation take up much of their time. Working conditions and patient load may be areas of further investigation to address this time strain and improve patient satisfaction [12]. 

For some general medicine clinics, patients surveyed expressed a lower satisfaction rating as time passed, which could be due to additional time to consider the visit and their health outcome or difficulty expressing dissatisfaction directly to the provider [13, 14]. Another study found that patient ratings were higher at two weeks and three months after a clinic visit, mainly due to symptom improvement [15]. Other factors that should be interpreted with the date of collection include gratitude for care at the time and patients’ ability to evaluate their satisfaction with post-discharge instructions and results [16]. Our study reflects similar results, demonstrating positive sentiment from patients awaiting discharge. Although this score is expected to drop with time, patient satisfaction at any period is still a valuable measure for performance, and interviewing immediately at discharge can reveal major concerns. 

Several limitations exist to this study. The challenge of utilizing a convenience sample is that, although the patients who consented to being interviewed provided positive input, we do not know the responses of attendees of the discharge lounge who did not consent to be interviewed. Although reported hospitalization data showed a variety of system reasons, it may not be a representative sample of the hospital population overall. This was designed as a pilot study without collecting specific patient data, which does limit analysis relating to demographics or conditions. Future studies could benefit from analyzing discharge understanding and patient outcomes in addition to demographic data. This study’s purpose was to understand the patient’s experience, with a view to expanding to more targeted, longitudinal, in-depth studies following patient outcomes after discharge. The sample size was small and limited to a single institution, thereby limiting generalizability to other institutions with different demographics or population data. Finally, patient satisfaction as a metric has been shown to be variably related to numerous factors, such as the time of delivery of the survey and progression or resolution of illness. Although short-term satisfaction may be high, long-term follow-up and patient outcomes would more effectively assess discharge process success. Specifically, readmission rates would be a key factor to study to confirm effectiveness. The qualitative feedback might suffer from interview bias or incomplete responses, as the survey was conducted orally. Patient responses may have been influenced by the interview setting and the presence of hospital staff, potentially limiting the honesty of the feedback.

Although there was a high reported understanding of hospital stay and discharge instructions among interviewed participants, perceived readiness does not necessarily equate to effective post-discharge care. A past study found that patient questionnaires completed directly after general surgery consultations had a higher satisfaction score than those completed later at home [14]. With time, degradation of knowledge can occur. Patients may have felt empowered at the time they were relayed information at discharge, but loss of information may occur by the time of follow-up. Still, patient perceptions and overall satisfaction are valuable markers in evaluating a hospital’s effectiveness and improving outcomes. Performing follow-up surveys on the patients from this study after a time lag would be beneficial to see if they follow the same trend. Patients may also demonstrate that the hospital’s discharge process is so robust that information is still retained. Determining which group these patients fall in can lead to either trying to increase this effective process or reevaluating the period between follow-ups. When standardizing institutional procedures, these metrics can be used to guide decisions and measure performance. Our results serve as an overall positive benchmark for our institution’s operations and patient outcomes, and they have also elucidated some areas that can be further investigated to determine if our patients’ high satisfaction is truly associated with better outcomes. Our study adds to the literature that effective care team communication is paramount to the success of patients after discharge. More physician engagement suggests patients feel more comforted and heard. Delays in discharge were also noted by patients, and fishbone analysis of reasons for delay could greatly improve discharge process time and quality. However, this is still an investigative project, with the primary aim to identify any existing needs in the current discharge process and where specifically care may be lacking. 

Meanwhile, our qualitative data demonstrate how patients, despite the chaotic nature of a hospital stay that includes anxiety, increased family member involvement, and the disruption of daily routine, can still provide valuable feedback on clinical processes. Particularly relevant are the positive sentiments surrounding the care team’s attitudes, demonstrating how small actions by hospital employees make a large impact. An important takeaway from both the qualitative responses and the quantitative data is that coordinating patient flow and discharge with adequate follow-up while still maintaining efficiency and high-quality care remains a difficult challenge to solve. 

## Conclusions

Hospital discharge is a complicated process that plays a role in patient readmission. The purpose of this study was to utilize patient interviews to assess satisfaction and perceived understanding of instructions as measures of discharge process performance. Findings indicate patients generally report positive experiences and preparedness for their subsequent care steps. Physician communication time was significantly related to satisfaction, while care team attentiveness and hospital flow were themes that arose from the interviews collected. Overall, high ratings from interviewed patients represent a positive sign for hospital discharge. However, recurring themes of logistical challenges and discharge delays are also a key part of the patient experience. Optimizing these aspects can help the effectiveness of the overall discharge process as well. Further study on demographics and follow-up for readmission can better assess discharge success. Continuing work analyzing readmission rates and similar metrics can lead to targeted future quality improvement initiatives. From this exploratory work, more robust studies, including the collection of demographic data and long-term care outcomes, can reveal any gaps in understanding as well as significant processes that aid in effective discharge. Ultimately, introducing interventions and policy changes because of these studies could improve patient outcomes. 

## Appendices

Appendix A

Survey Questions

General Visit/Medical Questions

What was the reason for your hospitalization?

Do you have a PCP and/or any specialists you follow up with on a regular basis?

Quantitative Questions 

Using the scale of: strongly disagree, disagree, neutral, agree, or strongly agree, answer: 

My questions were well answered prior to my discharge.

I understand what appointments/follow-ups I need to go to next.

I understand how to take my medications once I leave the hospital.

I am confident that I’ll be able to follow my discharge instructions at home. 

I understand my diagnosis, or the problem for which I stayed in the hospital. 

I feel prepared to leave the hospital.

I had adequate time talking to my physician during my hospital stay. 

I would return to Tampa General Hospital for future healthcare needs.

Lastly, on a scale of 0-10, how would you rate the quality of your care at your visit?

Qualitative Questions

Did you feel like you participated in your discharge process? Why or why not?

Do you feel ready to be discharged? If not, what support do you feel like you won’t have enough of at home?

Were there any parts of the hospital stay that were confusing or hard to understand?

How would you restructure the discharge process to best help future patients?

If a low rating on the fifth quantitative question: What would you like to understand more? 

What would you have liked the care team to focus on more prior to discharge?

Do you feel like your health care team that took care of you was cohesive and non-repetitive with your care?

What are your thoughts about the plan you and the doctor came up with prior to discharge?

Do you think that Telehealth would decrease any of your issues/obstacles in regards to your care?
